# Stability and Spin Waves of Skyrmion Tubes in Curved FeGe Nanowires

**DOI:** 10.3390/nano14181468

**Published:** 2024-09-10

**Authors:** Miguel-Angel Garrido-Tamayo, Eduardo Saavedra, Carlos Saji, Ulises Guevara, Laura M. Pérez, Liliana Pedraja-Rejas, Pablo Díaz, David Laroze

**Affiliations:** 1 Escuela de Física, Universidad Nacional de Colombia, Medellín 050034, Colombia; magarridot@unal.edu.co; 2 Departamento de Física, Universidad de Santiago de Chile (USACH), Santiago 9170124, Chile; 3 Departamento de Física, Facultad de Ciencias Físicas y Matemáticas, Universidad de Chile, Santiago 8370449, Chile; carlos.saji@ing.uchile.cl; 4 Instituto de Alta Investigación, Universidad de Tarapacá, Casilla 7D, Arica 1000000, Chile; uguevara@academicos.uta.cl (U.G.); dlarozen@academicos.uta.cl (D.L.); 5 Departamento de Ingeniería Industrial y de Sistemas, Universidad de Tarapacá, Casilla 7D, Arica 1000000, Chile; lperez@academicos.uta.cl (L.M.P.); lpedraja@academicos.uta.cl (L.P.-R.); 6 Departamento de Ciencias Físicas, Universidad de La Frontera, Casilla 54-D, Temuco 4811230, Chile; pablo.diaz@ufrontera.cl

**Keywords:** skyrmion tube, curvature, dynamic susceptibility, magnetic nanowires, micromagnetism, micromagnetic simulation, Mumax3

## Abstract

In this work, we investigate the influence of curvature on the dynamic susceptibility in FeGe nanowires, both curved and straight, hosting a skyrmionic tube texture under the action of an external bias field, using micromagnetic simulations. Our results demonstrate that both the resonance frequencies and the number of resonant peaks are highly dependent on the curvature of the system. To further understand the nature of the spin wave modes, we analyze the spatial distributions of the resonant mode amplitudes and phases, describing the differences among resonance modes observed. The ability to control the dynamic properties and frequencies of these nanostructures underscores their potential application in frequency-selective magnetic devices.

## 1. Introduction

During the last decade, magnetic nanowires have been studied due to their potential technological applications, highlighting their use as information storage units and in biomedical applications [1,2,3,4,5]. Early studies focused on straight nanowires made from ferromagnetic materials, exploring how geometry influences magnetic properties such as anisotropy and domain wall propagation [6,7,8].

A new advance in the topic has been incorporating chiral magnetic materials in manufacturing nanowires. These materials present the so-called Dzyaloshinskii–Moriya interaction (DMI), where spin–orbit coupling leads to asymmetric exchange interactions, causing a twisting of magnetic moments [9,10]. The presence of the DMI leads to the formation of magnetic skyrmions, which are topologically stable spin textures characterized by swirling arrangements of magnetic moments [11,12]. Skyrmions can be of several types, with Néel and Bloch skyrmions being the most common [13]. The application of an external magnetic field, the crystal symmetry of the material, and the DMI significantly influence the formation and stability of skyrmions [14,15,16]. An interesting review of the topic and its perspectives can be found in [17]. Among the materials that show DMI, one that stands out is iron monogermanide (FeGe), a non-centrosymmetric cubic helimagnet (space group $P2_{1}3$, B20-type structure) [18,19,20,21]. This material can be formed at atmospheric pressure and shows skyrmions at temperatures close to room temperature [22,23], making it an excellent prototype for studying chiral properties in nanostructures. Several experimental works [24,25,26] and micromagnetic simulations [27,28,29,30] have been carried out on cylindrical FeGe nanostructures, in which various chiral textures have been observed.

On the other hand, in recent years, several works have investigated how the curvature of nanowires and nanotubes affects these magnetic properties, such as anisotropy and dynamic susceptibility [31,32,33]. An interesting result mentioned in [31], a work in which curved Permalloy nanotubes were simulated, is that the curvature induces an effective DMI (even when the material used does not present DMI, as in this case). This point is relevant since, to date, curved nanowires based on materials that present DMI have yet to be explored, which represents a gap to explore.

Following this idea, in this study, we examine the static and dynamic responses of skyrmion tubes to external magnetic fields in both curved and straight nanowires. We have chosen FeGe as a base material due to its experimental accessibility. Moreover, in this material, the skyrmions have an approximate diameter of 70 nm [22], a size that allows them to be confined within nanowires with diameters of the order of 100 nm. This aspect is significant, as demonstrated in [34], as it means that skyrmions can traverse the material in the form of tubes, thereby making it possible to contain them within a nanowire. We determine the relaxed states in these curved geometries through numerical simulations under various external magnetic fields. Additionally, we identify the resonance frequencies and dynamic susceptibilities. The results demonstrate that the nucleation and stability of skyrmions are intrinsically dependent on geometry. Furthermore, we analyze the resonance modes of these magnetization patterns, illustrating how geometry influences the suppression of certain spin wave frequencies. By elucidating the interaction between curvature, topology, and magnetization dynamics, this study aims to understand the properties of skyrmions, a fundamental step toward the development of new applications based on their intriguing magnetic behavior [11,35], characterized by their topologically protected stability, nanoscale size, and non-trivial topology.

The manuscript is organized as follows: Section 2 shows the geometry construction and micromagnetic simulation of the nanotubes. Section 3 and Section 4 detail the results obtained and the conclusions.

## 2. Micromagnetic Simulations

To clarify the effect of geometry on the skyrmion tube stability and field-driven dynamics, we examine two systems: curved and straight nanowires. To analyze the spatiotemporal dynamics of the magnetization field, we solve the Landau–Lifshitz–Gilbert (LLG) equation, which has been widely studied [36,37,38,39,40,41]. The LLG equation is defined as

(1) $$\frac{d\vec{m}}{dt}=-\frac{\gamma }{1+\alpha^{2}}\left[\vec{m}\times {\vec{H}}_{\mathrm{eff}}+\alpha \vec{m}\times \left(\vec{m}\times {\vec{H}}_{\mathrm{eff}}\right)\right]\;$$

where α and γ represent the Gilbert damping constant and the gyromagnetic ratio, respectively. Here, $\vec{m}=\vec{M}/M_{s}$ denotes the normalized magnetization vector, and ${\vec{H}}_{\mathrm{eff}}=-\left(1/\mu_{0}M_{s}\right)\delta \varepsilon /\delta \vec{m}$ is the effective field, with $\varepsilon =\varepsilon_{ex}+\varepsilon_{d}+\varepsilon_{DM}+\varepsilon_{Z}$ being the total energy density, such that $\left(\varepsilon_{ex},\varepsilon_{d},\varepsilon_{DM},\varepsilon_{Z}\right)$ are the exchange, dipolar, Dzyaloshinskii–Moriya (DM), and Zeeman contributions, respectively. Therefore, the total energy density can be cast in the following form:

(2) $$\begin{array}{c} \varepsilon =A\left[{\left(\vec{\nabla }m_{x}\right)}^{2}+{\left(\vec{\nabla }m_{y}\right)}^{2}+{\left(\vec{\nabla }m_{z}\right)}^{2}\right]+\frac{1}{2}\mu_{0}M_{s}\left(\vec{m}\cdot \vec{\nabla }U\right)+D\vec{m}\cdot \left(\vec{\nabla }\times \vec{m}\right)-\mu_{0}M_{s}\vec{m}\cdot \vec{H}, \end{array}$$

where *A*, $\mu_{0}$, $M_{s}$, and $D_{b}$ are the exchange constant, vacuum magnetic permeability, saturation magnetization, and DM interaction constant, respectively. Furthermore, the term *U* is the scalar magnetic potential, which is defined as

$$\begin{array}{c} U=\frac{1}{4\pi }\oint_{S}\frac{{\vec{n}}^{\prime }\cdot {\vec{M}}^{\prime }}{|{\vec{r}}^{\prime }-\vec{r}|}\;dS^{\prime }-\frac{1}{4\pi }\int_{V}\frac{{\vec{\nabla }}^{\prime }\cdot {\vec{M}}^{\prime }}{|{\vec{r}}^{\prime }-\vec{r}|}\;dV^{\prime }, \end{array}$$

with ${\vec{n}}^{\prime }$ as the vector normal to the surface, ${\vec{r}}^{\prime }$ and $\vec{r}$ are the position vectors, *S* is the surface of the volume considered, and *V* is the volume of the magnetic material.

For all our calculations, we consider that the material parameters are an exchange stiffness $A=4.75\times 10^{-12}$ J m^−1^ and a saturation magnetization $M_{s}=0.384\times 10^{6}$ Am^−1^, and a DMI constant $D_{b}=0.853\times 10^{-3}$ J m^2^ corresponding to FeGe [25,26]. To compute numerically the LLG equation, we use the Mumax3 software [42], which has been vastly employed in several systems [43,44,45,46,47,48,49,50,51,52]. This software uses a finite difference discretization method. Therefore, to simulate with a curved system, the unit cell must be small. In particular, we have considered unit cell sizes of 2×2×2 nm^3^ to describe a smooth geometry and to neglect border effects [53], additional calculations were performed with 1 × 1 × 1 nm^3^ cells for n=6, but no appreciable changes were observed. Micromagnetic simulations were conducted on a computer system optimized with an GeForce RTX® 3090 Ti GPU featuring 10752 CUDA cores by Nvidia corporation in EEUU. This configuration provides exceptional computational power, rendering it one of the premier choices for executing GPU-accelerated micromagnetic research [48].

The construction of a curved nanowire on an xz plane follows the same rules of construction of a nanowire with toroidal geometry under certain conditions; the vector with the parametric equations is described below:

(3) $$\vec{r}=\left[R_{T}-r_{p}cos\left(\theta \right)\right]cos\left(\phi \right)\hat{x}+\left[R_{T}-r_{p}cos\left(\theta \right)\right]sin\left(\phi \right)\hat{z}$$

The geometric parameters that describe the nanowire with the toroidal geometry on the xz plane are as follows. $R_{T}$ represents the toroidal radius measured from the origin of the coordinate system, $r_{p}$ is the poloidal radius that describes the thickness of the toroid, θ represents the poloidal angle of the toroid, and ϕ is the azimuthal angle or scanning angle on the xz plane. *L* is an arc length that remains constant at 1000 nm. The toroidal radius and the azimuthal angle of the xz plane vary depending on the following relationship:

(4) $$\phi =\frac{2\pi }{n};\;R_{T}=\frac{nL}{2\pi }.$$

**Figure 1:**
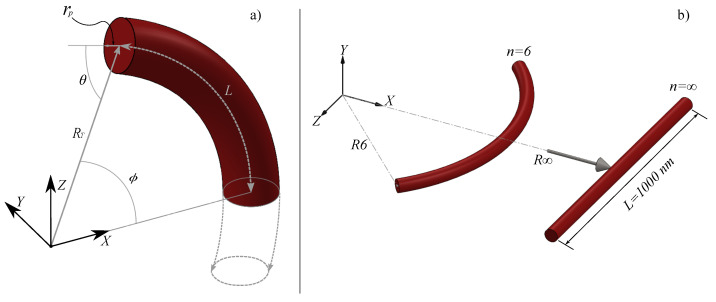
(**a**) Geometrical parameters define the system. $R_{T}$ represents the toroidal radius, $r_{p}$ is the poloidal radius, θ is the poloidal angle of the toroid, and ϕ is the azimuthal angle or angle on the xz-plane. *L* is the constant arc length of 1000 nm. (**b**) Sketch representing some of the different NWs considered in this work. The label n identifies the curvature of the NW. The curvature radius for each NW (Rn) is depicted with arrows. All NWs have a length of 1000 nm and a diameter of 100 nm.

When n=6, the nanowire describes a curvature that represents one-sixth of a circle; when n=∞, the nanowire could be considered as a segment without curvature, that is, a straight segment (see Figure 1b). All NWs have a length of 1000 nm and a diameter of 100 nm.

Previous research has demonstrated that the stable or metastable state of isotropic chiral magnetic nanowires comprises skyrmion tubes [26,54,55]. Following this line of inquiry, a two-step process was initially implemented to attain this relaxed state for different external magnetic fields, $\vec{H}=H\hat{z}$, where the LLG equation was numerically solved for the entire system, maintaining a maximum torque exceeding [56,57] $10^{-4}$ T, with a damping parameter set at α = 0.5. Subsequently, a time minimization step lasting 100 nanoseconds was conducted to mitigate spin waves associated with magnetization relaxation [58]. The dynamical response of the magnetization is obtained including a sinc field, $\vec{h}\left(t\right)=h_{0}\;$ sinc$\left[2\pi f_{\max}\left(t-t_{0}\right)\right]\;\hat{z}$ [31,59]. Here, the amplitude is set at $h_{0}=1$ mT, with a cut-off frequency of f=25 GHz. The spatial profile of the *x*-component of the magnetization was saved every 20 ps for a total simulation time of 100 ns, and the frequency resolution is 0.01 GHz. In this process, we used a lower damping constant α=0.008 [31,60], which provides a better resolution of the spin wave modes. Using the Fast Fourier Transform (FFT), we transformed the magnetic pulse, $\vec{h}\left(t\right)$, and the magnetization $\vec{M}$, to the frequency domain $\left[\vec{h}\left(\omega \right),\vec{M}\left(\omega \right)\right]$, respectively. The dynamic susceptibility ($\chi^{\prime \prime }$) was computed by dividing the Fourier transform of the response $\vec{M}\left(\omega \right)$ by the transform of the magnetic pulse $\vec{h}\left(\omega \right)$ [61,62].

## 3. Results

**Figure 2:**
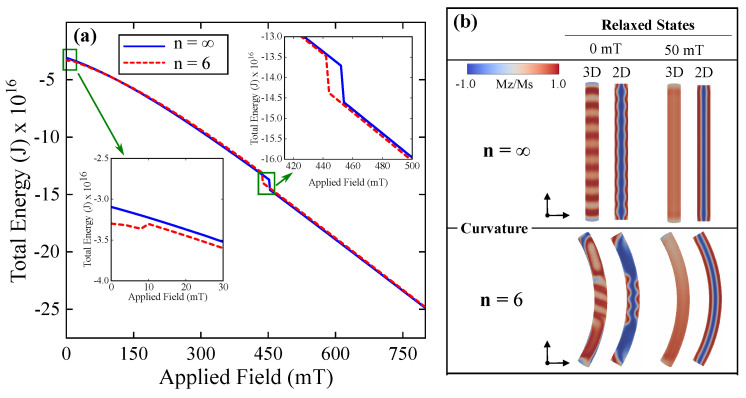
(**a**) Magnetic energy after relaxation from the initial skyrmion tube configuration as a function of the applied field for curved (n=6) and straight (n=∞) nanowires. (**b**) Magnetic configurations post-relaxation for curved (n=6) and straight (n=∞) nanowires at 0 mT and 50 mT, shown from different perspectives: the complete 3D structure and a 2D cross sectional view in the z plane of the structure. Colors represent the direction of magnetization: magnetization pointing upwards is shown in red, magnetization pointing downwards in blue, and magnetization pointing to the right or left in white (For an interpretation of the colors in this figure legend, please refer to the web version of this article).

**Figure A1:**
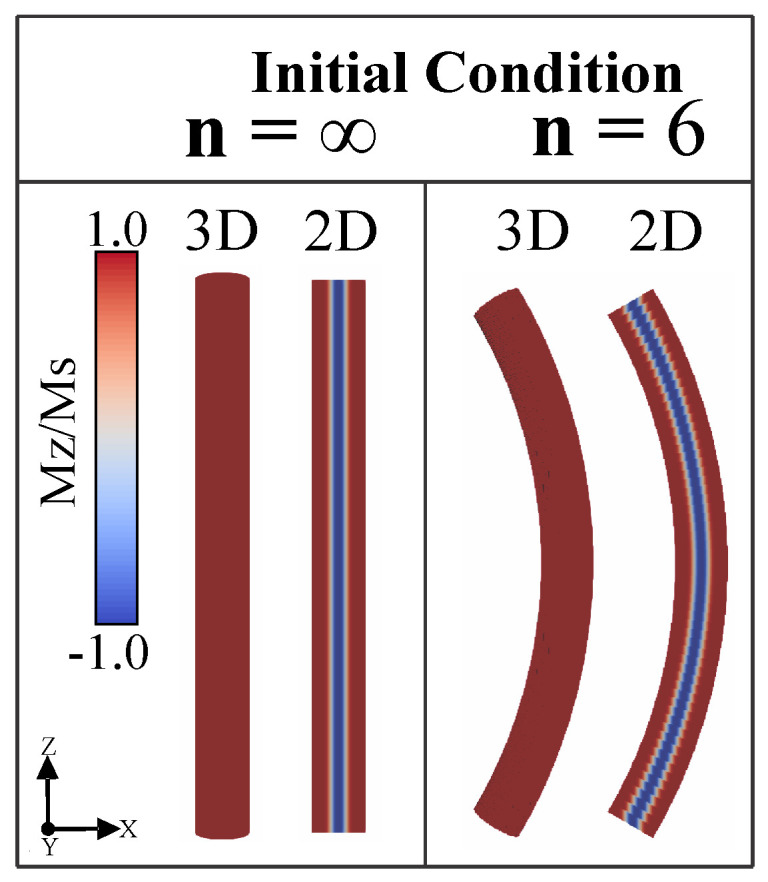
Illustration of the configuration initially inscribed. Here, we present a skyrmion tube configuration: the core of the nanostructure points its magnetization downwards (blue), while the surface points its magnetization upwards (red) (For an interpretation of the colors in this figure legend, please refer to the web version of this article).

**Figure A2:**
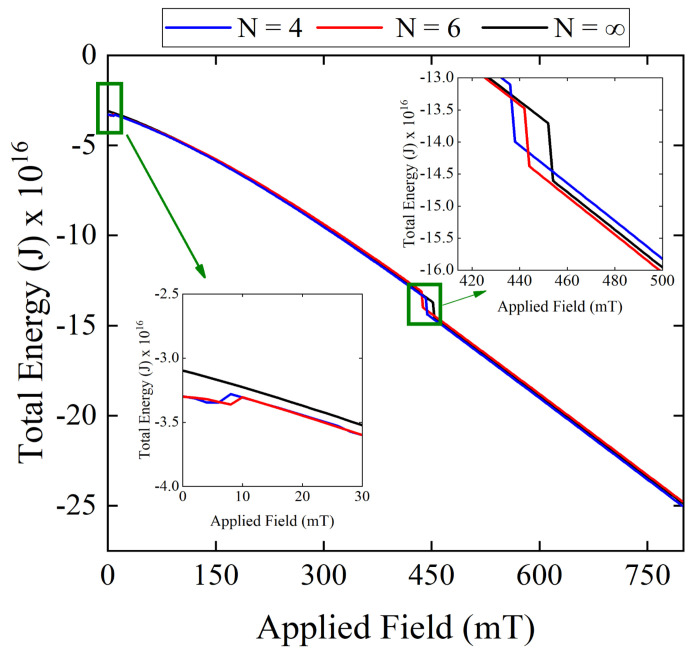
Magnetic energy after relaxation from the initial skyrmion tube configuration as a function of the applied field for curved (n=4), (n=6) and straight (n=∞) nanowires (For an interpretation of the colors in this figure legend, please refer to the web version of this article).

We start our simulations with an initial configuration in which the magnetization is a skyrmion tube (see Figure A1 and code of initial configuration in Appendix A). Once the skyrmion tube is hosted in curved and straight structures, we address its response to a static magnetic field to understand how the geometry affects the skyrmion tube stability. Therefore, we apply a magnetic field $\vec{H}=H\hat{z}$ varying from 0 to 800 mT along the z-axis. The energy as a function of the external magnetic field is then obtained and presented in Figure 2a. The figure shows that for the straight nanowire (n=∞), two distinct regions of magnetization profiles are stabilized, separated by the threshold magnetic field value of *H* = 452 mT. Below this threshold, the magnetization corresponds to a skyrmion tube, while above 452 mT, the magnetization predominantly aligns with the z-direction. In contrast, for the curved nanowire (n=6), three different magnetic field regions are observed, each with distinct magnetization profiles ranging from 0 mT to 10 mT. A helical state is present within this range, followed by a skyrmion tube until 442 mT. Beyond this point, the magnetization predominantly aligns with the z-direction for n=6, extending up to 800 mT. We observe that *n* = 4 (see Figure A2 in Appendix A) exhibits behavior very similar to n=6, with three distinct regions of the magnetic field, each corresponding to different magnetization profiles ranging from 0 mT to 6 mT. Within this range, a helical state is present, followed by a skyrmion tube up to 438 mT. Beyond this point, the magnetization predominantly aligns with the z-direction, extending up to 800 mT. To better describe the observed states, we analyze the magnetization in the external surface and the cross-sections along the z-axis direction, presented, respectively, in Figure 2b.

In order to better understand the topologically non-trivial nature of our magnetization configuration and confirm the origin of the skyrmion tube annihilation, we have calculated the value of the 2D topological charge for different cross-sections across the nanostructure length according to the following equation:

(5) $$Q=\frac{1}{4\pi }=\int_{S}\vec{m}\cdot \left(\frac{\partial \vec{m}}{\partial x}\times \frac{\partial \vec{m}}{\partial y}\right)\;dx\;dy$$

**Figure A3:**
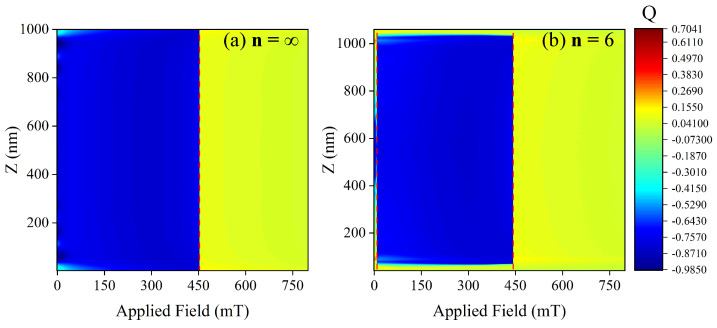
A 2D topological charge for different cross-sections across the nanostructure length: (**a**) straight nanowire and (**b**) curved nanowire under the action of an external magnetic field (For an interpretation of the colors in this figure legend, please refer to the web version of this article).

Although the widely used concept of the topological charge (skyrmion number or degree of mapping) in 2D spin systems is not directly applicable to our nanowires because the magnetization depends on the thickness coordinate z, m(*x*, *y*, *z*), several works in the literature report 2D topological charge analysis in the function of z dimension [53,55,63]. Continuing along this line of thought, we have determined the skyrmion number of each cross-section along the z-axis direction for the length of each nanostructure for n=∞ and n=6 under the action of an external magnetic field. Our findings are illustrated in Appendix A in Figure A3, revealing a sudden change in the skyrmion number when the critical field separating the two or three states is reached. For *n* = *∞*, when H≤ 452 mT, the topological charge is Q ≈ −1, while for H> 452 mT, we observe a value of Q ≈ 0, indicating that this configuration is topologically equivalent to a ferromagnetic state. On the other hand, for n=6, at 10 mT ≤H≤ 442 mT, the topological charge is Q ≈ −1, while for H> 442 mT, we obtain Q ≈ 0.

### 3.1. Dynamic Susceptibility for Straight Nanowires (n=∞)

**Figure 3:**
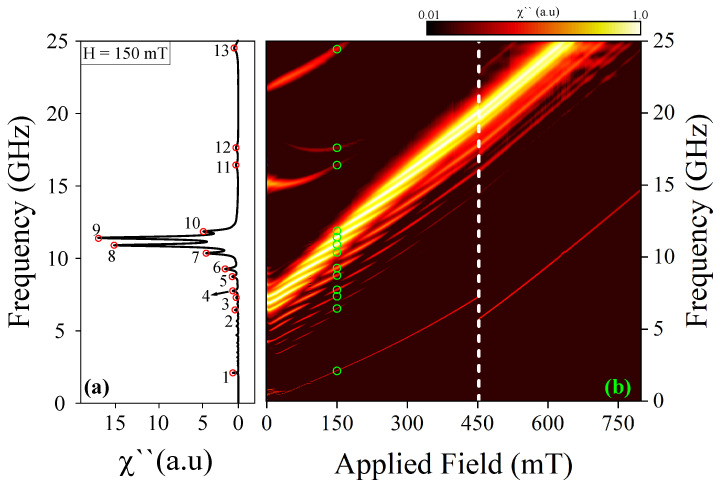
(**a**) Spin wave resonant spectrum at 150 mT for straight nanowires and (**b**) frequency of the resonant modes as a function of the applied field. The color map is presented on a logarithmic scale, from 0.01 to 1.0.

We now explore the frequency modes of the magnetic skyrmion tube using the sinc field mentioned above. In this scenario, the magnetic field triggers resonant modes contingent upon the magnetic configuration of the nanostructure with *n* = *∞*. It is noteworthy that the amplitude of the pulse is sufficiently small to ensure that the magnetic response of the system remains within the linear regime [64,65]. The findings are depicted in Figure 3b, which showcases the resonance mode frequencies as a function of $\vec{H}=H\hat{z}$. The results indicate that the resonant modes are contingent upon the external magnetic field, with changes in the spectrum of resonant modes correlating to the annihilation of the skyrmion tube state and the nucleation of the uniform state along the z-direction. Importantly, it is observed that when the system hosts a skyrmion tube state (at *H* = 150 mT), thirteen resonant modes can be observed, here called modes 1 to 13, each corresponding to a resonant peak shown in Figure 3a. Several studies have focused on identifying and simplifying modes by emphasizing the most prominent ones [30,66,67]. In this work, we adopted a similar approach by concentrating on the most significant modes at a magnetic field of *H* = 150 mT. This allowed us to offer a more detailed yet simplified description of the observed phenomena.

**Figure 4:**
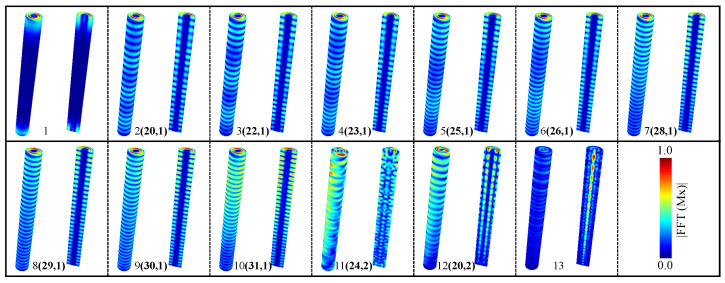
Spatial distribution of the excited spin wave modes when applying a small microwave magnetic pulse to a straight nanowire. The color code establishes the amplitude of the FFT employed on the x-component of the magnetization field. The number pairs (Nv, Nr) are Nv, antinodes along the vertical direction and Nr, nodes along the radial direction. The red color implies a higher spin amplitude, while the blue color means a zero spin amplitude.

To better understand the spatial nature of the SW resonance modes, we obtained the spatial distribution of the amplitudes corresponding to the spectra of the resonant modes from numerical calculations. For this purpose, we determined the Fourier image of each magnetic moment $m\left(r_{ijk},\omega_{n}\right)=\mathrm{DFTt}\left(m\left(r_{ijk},t_{n}\right)\right)$ (obtained from Mumax3 simulations) where DFTt is the Fourier transform. The subscripts ijk correspond to each cell’s spatial coordinates *x*, *y*, and *z*. In contrast, the subscript *n* denotes the frequency position of the power spectrum. We investigate the characteristics of SW (spin wave) modes through numerical simulations, analyzing the spatial distribution of amplitudes and phases of resonances. As illustrated in Figure 4, our results depict the spatial modes of the frequency spectrum within the skyrmion tube state for *H* = 150 mT, hosted in a straight nanowire. We categorize the observed modes into four primary groups: (a) low-frequency modes (mode 1), characterized by perturbations primarily originating from magnetization excitations at the borders of the nanostructure; (b) low–intermediate-frequency modes (from mode 2 to mode 10), exhibiting perturbation patterns predominantly near the surface with a single-radial node; (c) high–intermediate-frequency modes (mode 11 and mode 12), displaying perturbation patterns near the surface with double radial nodes; and (d) high-frequency modes (mode 13), with perturbations primarily localized at the center of the magnetic structures (excited skyrmion tube state). Distinguishing between low-, intermediate-, and high-frequency modes is achieved by observing the number of antinodes and nodes present in their resonant profiles on the surface and along the radial zone. To simplify this analysis and describe the characteristics of each eigenmode, we define a set of number pairs (Nv, Nr). Nv describes the number of antinodes along the vertical direction [29], while Nr indicates the number of nodes along the radial direction [68].

### 3.2. Dynamic Susceptibility for Curved Nanowires (n=6)

**Figure 5:**
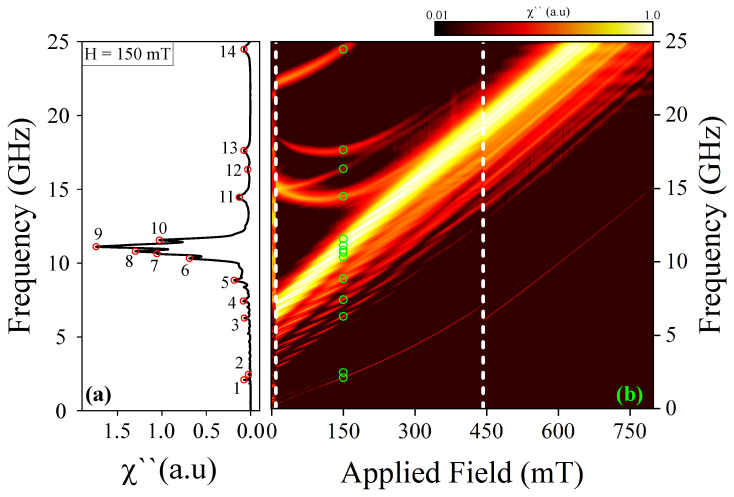
(**a**) Spin wave resonant spectrum at 150 mT for curved nanowire and (**b**) frequency of the resonant modes as a function of the applied field. The color map is presented on a logarithmic scale, from 0.01 to 1.0.

For the sake of completeness and comparison, we now delve into the dynamic response of magnetization within the magnetic configuration of the nanostructure with n=6. The results are illustrated in Figure 5. Similar to the case with n=∞, our findings suggest that the resonant modes are contingent upon the external magnetic field, whereby alterations in the spectrum of resonant modes correspond to the annihilation of the skyrmion tube state and the nucleation of the uniform state along the z-direction. Notably, the effect of curvature is observed when the system hosts a skyrmion tube. At *H* = 150 mT, where the system exhibits a skyrmion tube state, fourteen resonant modes are observable, herein referred to as modes 1 to 14, each corresponding to a resonant peak as depicted in Figure 5a.

**Figure 6:**
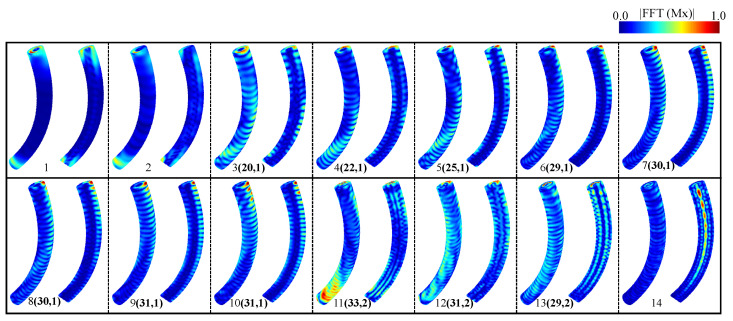
Spatial distribution of the excited spin wave modes when applying a small microwave magnetic pulse to a curved nanowire. The color code establishes the amplitude of the FFT employed on the x-component of the magnetization field. The number pairs (Nv, Nr) are Nv antinodes along the vertical direction and Nr nodes along the radial direction. The red color implies a higher spin amplitude, while the blue color means a zero spin amplitude.

**Figure A4:**
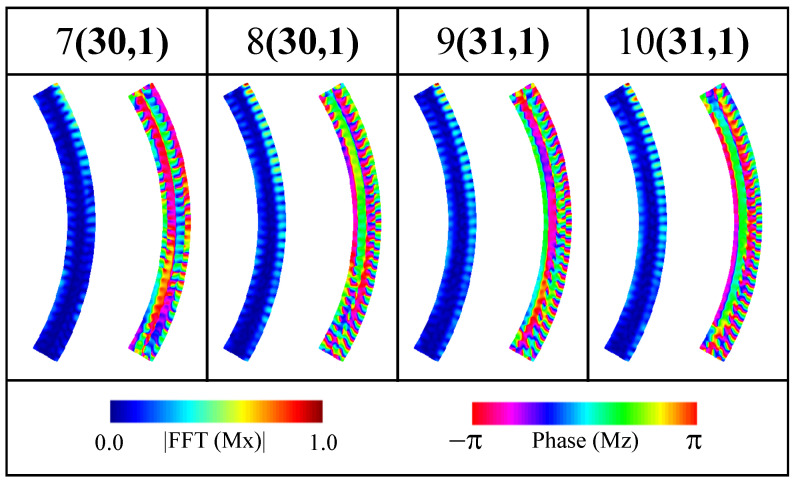
(Left of each panel) The x-component of the excited magnetization field for four frequency modes, respectively. (Right of each panel) The z-component spectral phase of the dynamic susceptibility for each corresponding resonance frequency. All cases correspond to curved nanowires (n=6). The number pairs (Nv, Nr) are Nv antinodes along the vertical direction and Nr nodes along the radial direction (For an interpretation of the colors in this figure legend, please refer to the web version of this article).

In a similar manner, we investigate the spatial distribution of resonance amplitudes. As depicted in Figure 6, our findings elucidate the spatial modes of the frequency spectrum within the skyrmion tube state for *H* = 150 mT, situated in a curved nanowire. Once more, these modes are observed to cluster into four primary groups. In this instance, the low-frequency modes correspond to modes 1 and 2, the low–intermediate-frequency modes encompass modes 3 to 10, the high–intermediate-frequency modes pertain to modes 11 and 13, and finally, the high-frequency modes correspond to mode 14. It is important to note that among the observed resonant modes, modes 7 and 8 share the same number of vertical antinodes (30 antinodes) and radial nodes (1 node), while modes 9 and 10 have 31 vertical antinodes and 1 radial node. To differentiate between these modes, we have determined the phase of the excited magnetic moments, allowing us to explore the activation mechanisms of these modes. From Figure A4 in Appendix A, which represents the phase of the Fourier analysis along the z-axis for each mode, we can deduce distinct behavioral characteristics. It is important to note that when a straight nanowire (*n* = *∞*) hosts a skyrmion tube, its frequency activates from 21.77 GHz to 24.92 GHz within a magnetic field range of 0 mT to 176 mT. On the other hand, the skyrmion tube excitation mode for a curved nanowire (n=6) activates from 22.17 GHz to 24.87 GHz within a magnetic field range of 10 mT to 176 mT. Additionally, in the high–intermediate frequencies, a new resonant mode was identified in the curved nanowire that does not appear in the straight nanowire, with frequencies decreasing from 15.28 GHz to 14.27 GHz between 10 mT and 110 mT, and then increasing to 17.23 GHz at 324 mT, which may be a result of the curvature. In the low–intermediate frequencies, there is closer proximity among the peaks of greater dynamic susceptibility for the curved nanowire, and a higher number of resonant modes were observed between 10.00 GHz and 12.00 GHz, which corresponds to the frequency range of the highest magnetic susceptibility for a 150 mT field in both cases. In this range, the curved nanowire exhibits its highest susceptibility at 11.10 GHz, unlike the straight nanowire, which shows two peaks around 11.00 GHz.

Finally, to interpret the magnetization dynamics, we must consider that the skyrmion tube has three zones: an external ferromagnetic cylindrical region connected to an intermediate vortex-type configuration and a ferromagnetic cylindrical core anti-aligned with the external cylindrical region. The DMI topologically protects this structure. The magnetic field pulse along the x-axis perturbs the xy components of the magnetization within the defined frequency range, generating oscillations that are transmitted through the nanowire.

**Figure A5:**
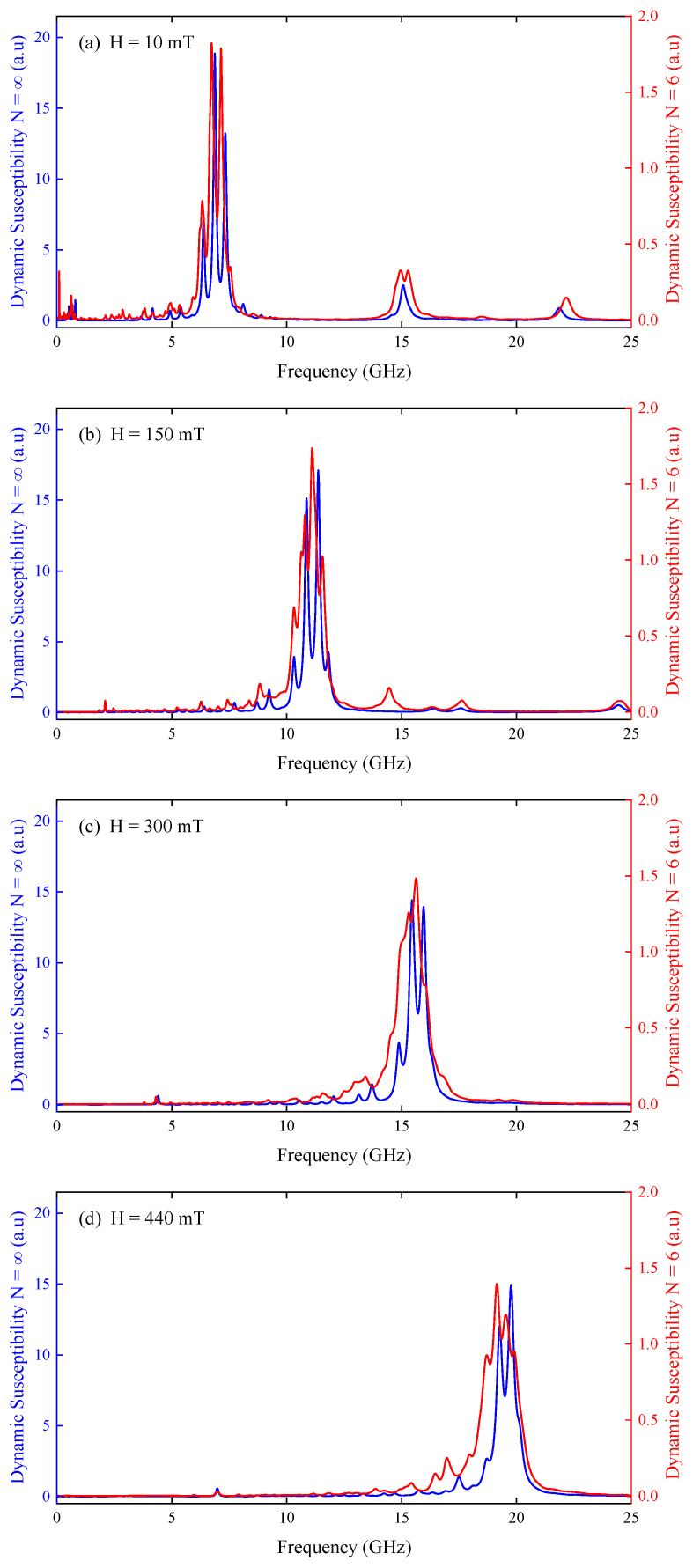
Frequencies at different values of the applied magnetic field (For an interpretation of the colors in this figure legend, please refer to the web version of this article).

As mentioned, in the case of the straight nanowire, at low and intermediate–low frequencies, the oscillation pattern includes a standing wave along the z-axis in the external cylindrical region, with a radial node in the shape of a ring. These oscillations do not propagate towards the core. We observe that the oscillations are more intense in the external cylindrical region due to the greater exposure of the nanowire surface and fewer constraints compared to the internal regions, which facilitates the development of magnetic oscillations in this region. Additionally, exchange interactions and surface anisotropy may promote the propagation of spin wave modes in the outer layer. The internal structure of the nanowire, including vortex-like configurations or an anti-aligned ferromagnetic core, tends to act as a barrier to the propagation of oscillations from the surface toward the interior. However, at higher resonant frequencies, the oscillations are primarily concentrated in the core, albeit with lower amplitude. In the case of the curved nanowire, the symmetry breaking prevents the formation of well-defined patterns and leads to a broadening of the frequency peaks (see Figure A5). On the other hand, since the vortex-like structures between the two ferromagnetic regions are not parallel to the x-axis, they lose their protective function over the core, allowing oscillations to penetrate it with greater intensity and across a wider range of frequencies, as observed in the high- and intermediate–high-frequency peaks.

In summary, these results highlight that the skyrmion tube structure allows for the generation of spin waves in the external cylindrical region without disturbing the core over a wide range of frequencies. The symmetry breaking affects both the spatial distribution of the resonant modes and the frequency response, resulting in a broader and more complex spectrum, with less-defined oscillatory patterns and more dispersed resonant energy across the different regions of the nanowire.

## 4. Conclusions

In this study, we have investigated the static and dynamic properties of magnetic nanowires in the context of variable curvature, with a specific focus on the formation and behavior of skyrmion tubes. The results indicate that the geometry of the nanowire significantly influences the transition of magnetic states and resonant dynamics. As for the static properties, our findings reveal varied magnetic state transitions, i.e., the straight nanowire shows two magnetic regions (the skyrmion tube and uniform regions), unlike the curved nanowire, which shows three magnetic regions: helical, skyrmion tube, and uniform Z regions.

From a dynamic perspective, it was observed that the amplitude of the magnetic susceptibility notably decreases in curved nanowires, a phenomenon attributable to the minimization of torque between the magnetization and the induced microwave field. Furthermore, an adjustment was detected in the resonant modes towards higher frequencies in skyrmion states as the magnetic field increased. In the high–intermediate frequencies, a new resonant mode was identified in the curved nanowire that does not appear in the straight nanowire. In the low–intermediate frequencies, there is closer proximity among the peaks of greater dynamic susceptibility for the curved nanowire, as a consequence of an increase in bandwidth.

These results suggest that the curvature of the nanowire not only modifies the magnetic state transitions but also affects the resonant dynamics, offering possibilities for manipulating magnetic responses in applications where precise frequency control is crucial. The variability observed in the resonant modes, particularly in curved nanowires, highlights the potential application of these nanostructures in frequency-controllable devices, taking advantage of the unique properties induced by the curvature of the material.

## Data Availability

Data will be made available on request.

## Appendix A

In this appendix, we provide additional details regarding the micromagnetic simulations employed for stabilizing the skyrmion tube. We delve into the analysis of the 2D topological charge as a function of the z dimension and elucidate the phase behavior of the excited magnetic moments across resonance modes, specifically focusing on modes 7 through 10 observed in curved nanowires. Additionally, we have included part of the Mumax3 code used to configure the curved nanowire and initialize the magnetization for the case of n=6, offering further insights into the setup and computational methods utilized in this study.

To attain equilibrium states, we initially solved the Landau–Lifshitz–Gilbert (LLG) equation for the complete system, employing a skyrmion tube magnetic configuration. Figure A1 illustrates the initially inscribed configuration. Specifically, we depict a skyrmion tube configuration wherein the core of the nanostructure exhibits magnetization pointing downwards (depicted in blue), while the surface displays magnetization pointing upwards (depicted in red). This configuration serves as the starting point for our investigation into the stabilization and dynamics of skyrmion tubes within the system.

This is part of the Mumax3 code with which the configuration of the curved nanowire and the initial magnetization for n=6 is performed:

		#####Part of code to define the geometry with n=6#####
		 
		angle:=angles*(pi/180)
		angle_:=(90+angles)*(pi/180)
         
		x := R_cur*sin(angle)
		y := 0
		z := R_cur*cos(angle)
		Cilin_T := Cylinder(d_mon, h_mon).roty(angle_)
		.transl(x,y,z).transl(-0.88*R_cur,0,0)
		istep := 1
		 
		for i:=0; i<=angles; i+=istep{
		 
		theta:=i*(pi/180)
		 
		x := R_cur*sin(angle+theta)
		y := 0
		z := R_cur*cos(angle+theta)
		Cilin := Cylinder(d_mon, h_mon).roty(angle_+theta).transl(x,y,z)
		.transl(-0.88*R_cur,0,0)
		Cilin_T=(Cilin_T.add(Cilin))
		}
		######Part of code to define the initial magnetization with n=6#####
		 
		for i:=0; i<=angles; i+=istep{
		 
		theta:= i*(pi/180)
		 
		x := R_cur*sin(angle+theta)
		y := 0
		z := R_cur*cos(angle+theta)
		m.setinshape(Cylinder(d_mon, h_mon).roty(angle_+theta).transl(x,y,z)
		.transl(-0.88*R_cur,0,0), BlochSkyrmion(1,-1)
		.transl(x,y,z).transl(-0.88*R_cur,0,0))}
		

Figure A2 shows the variation of the energy as a function of the applied magnetic field for the three nanowires studied.

Figure A3 shows a 2D topological charge for different cross-sections across the nanostructure length as a function of the z dimension for straight nanowires and curved nanowires under the action of an external magnetic field.

In Figure A4, we elucidate the phase behavior of the excited magnetic moments across resonance modes, specifically focusing on modes 7 through 10 observed in curved nanowires.

In Figure A5, we explain the behavior of the frequencies at different values of the applied magnetic field.
